# Clinical Variability of ADPKD in Monozygotic Twins

**DOI:** 10.1016/j.ekir.2025.11.002

**Published:** 2025-11-11

**Authors:** Othmane Mohib, Stefan Van Cauwelaert, Laura Pölsler, Patricia Van Der Niepen, Carola Brussaard, Bruno Van Vlem, Rachel Schauer, Peter C. Harris, Peter Janssens

**Affiliations:** 1 Department of Nephrology and Arterial Hypertension, Universitair Ziekenhuis Brussel, Brussels, Belgium; 2 Kidney Diseases, Dialysis and Transplantation Research Unit, Vitality Research Group, Vrije Universiteit Brussel, Brussels, Belgium; 3 Kidney Center, AZORG, Aalst, Belgium; 4 Clinical Sciences, Research Group Genetics, Reproduction and Development, Centre for Medical Genetics, Vrije Universiteit Brussel, Universitair Ziekenhuis Brussel, Brussels, Belgium; 5 Department of Radiology, Universitair Ziekenhuis Brussel, Brussels, Belgium; 6 Division of Nephrology and Hypertension, Mayo Clinic, Rochester, Minnesota, USA

**Keywords:** ADPKD, clinical variability, monozygotic twins

## Introduction

Autosomal dominant polycystic kidney disease (ADPKD) shows remarkable variability in clinical severity, even among individuals with the same *PKD1* or *PKD2* pathogenic variants. This variability likely reflects complex interactions between genetic, epigenetic, comorbidities, and environmental factors^1^ (Supplementary Figure S1).

Monozygotic (MZ) twins with ADPKD, who share the same pathogenic germline variant and have highly similar modifier genes and polygenic risk as well as early-life environment, provide a unique opportunity to disentangle the contributions of intrinsic biological factors and extrinsic influences in shaping the natural history of ADPKD. To better understand modifying influences, we combined a review of reported MZ twin cases with 3 newly characterized MZ twin pairs carrying identical *PKD1* or *PKD2* variants, who nonetheless showed marked intrapair phenotypical differences.

## Results

We identified 3 pairs of MZ twins with genetically confirmed ADPKD followed-up with at our institution. Monozygosity was confirmed by > 99% concordance in single nucleotide polymorphisms. Targeted next-generation sequencing panels covering 356 cystic kidney disease and ciliopathy genes were analyzed in all pairs. Clinical data were reviewed from diagnosis through to kidney failure or last follow-up (summarized in Table 1). A literature review identified 2 previous studies describing MZ twins with ADPKD.^2^^,^^3^ Detailed methods are provided in the Supplementary Methods and detailed genetic results are provided in the Supplementary Genetic Results.

**Table 1:** Clinical and genetic characteristics of the 3 ADPKD monozygotic twin pairs

Twin pair		1		2		3	
Genetics							
	Gene/variant summary	*PKD1* (c.2249G>A, p.(Trp750Ter), het, truncating)		*PKD1* (c.11343C>G, p.(Tyr3781Ter), het, truncating)		*PKD2* (c.2635del, p.(Glu879Argfs∗30), het, truncating)	
Demographics							
	Sex and age (yrs)	Female, 31		Male, 45		Female, 75	
	Subject	1A	1B	2A	2B	3A	3B
ADPKD							
	Age at diagnosis (yrs)	5	5	24	24	43	42
	Tolvaptan (age at initiation in yrs)	Yes (23)	Yes (23)	Yes (35)	Yes (28)	No (N/A)	No (N/A)
	eGFR CKD-EPI (ml/min per 1.73 m^2^) at tolvaptan initiation	86	114	45	121	N/A	N/A
	Mayo Class (htTKV [ml/m])	1E (609)	1E (630)	1E (1658)	1C/D (468)	N/A	N/A
	Age at KF (yrs)	N/A	N/A	39	44	60	58
	Last eGFR CKD-EPI (ml/min per 1.73 m^2^)	42	90	N/A	10	N/A	N/A
	Liver cysts/brain aneurysm	No/No	No/No	Yes/N/A	Yes/N/A	Yes/Yes	Yes/No
Hypertension							
	Age at diagnosis	14	14	14	24	N/A	N/A
	Age at treatment initiation (yrs)	15	15	24	24	N/A	N/A
	ACEi or ARB	Yes	Yes	Yes	Yes	N/A	N/A
Other							
	Obesity/smoking	Yes/No	Yes/No	Yes/Yes	Yes/No	No/No	Yes/No

ACEi, angiotensin- converting enzyme inhibitors; ADPKD, autosomal dominant polycystic kidney disease; ARB, angiotensin receptor blockers; eGFR CKD-EPI, estimated glomerular filtration rate based on the Chronic Kidney Disease Epidemiology Collaboration formula; het, heterozygous; htTKV, height-adjusted total kidney volume; KF, kidney failure; N/A, not applicable.

#### Twin Pair 1

**Figure 1:**
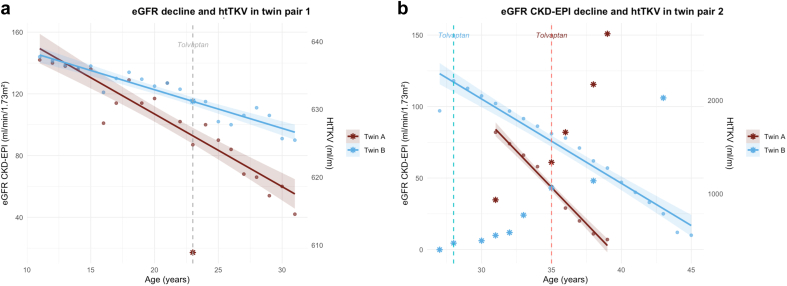
eGFR CKD-EPI trajectories and htTKV in (a) twin pair 1 and (b) twin pair 2. Dots represent individual eGFR measurements. Solid lines show the linear regression fit (95% confidence interval [CI] shaded area). Stars indicate htTKV values (right axis). Vertical dashed line = Tolvaptan initiation. Twin 1A showed a faster annual decline in eGFR CKD-EPI of −4.71 ml/min per 1.73 m^2^/yr (95% CI, −5.53 to −3.89) compared with −2.5 ml/min per 1.73 m^2^/yr (95% CI, −2.92 to −2.08) in twin 1B. Twin 2A showed faster eGFR CKD-EPI decline of −10.17 ml/min per 1.73 m^2^/yr (95% CI, −11.19 to −9.14) compared with −5.90 ml/min per 1.73 m^2^/yr (95% CI, −6.61 to −5.19) in twin 2B. eGFR CKD-EPI, estimated glomerular filtration rate based the Chronic Kidney Disease Epidemiology Collaboration formula; htTKV, height-adjusted total kidney volume.

Both 31-year-old women, born at 33 weeks because of preeclampsia, inherited a truncating *PKD1* variant from their mother, in a family with a history of kidney failure before the age of 50 years. They were diagnosed in childhood following cascade screening and developed arterial hypertension at the age of 14 years, which rapidly required triple antihypertensive therapy. Blood pressure control was consistently better in twin 1B. Both twins had similar body mass index trajectories and initiated tolvaptan at the age of 23 years, yet kidney function declined more rapidly in twin 1A (Figure 1a). The expected age of kidney failure using the Mayo Clinic Calculator is approximately 37 years for twin 1A and 43 years for twin 1B.

#### Twin Pair 2

These 45-year-old men carry a truncating *PKD1* variant. Arterial hypertension was diagnosed at the age of 14 years in twin 2A and at the age of 24 years in twin 2B. Both started antihypertensive therapy at age 24 years; twin 2A had better blood pressure control. Twin 2A, is a former smoker, developed obesity at a younger age, and started tolvaptan 7 years after twin 2B. Twin 2A reached kidney failure by the age of 39 years, whereas twin 2B reached kidney failure at 44 years (Figure 1b).

#### Twin Pair 3

Both 75-year-old women carrying a truncating *PKD2* variant reached kidney failure at the age of 58 and 60 years, respectively. They exhibited divergent extrarenal phenotypes. Twin 3A experienced a cerebral aneurysm rupture and required combined liver-kidney transplantation, in contrast to twin 3B, who showed no brain aneurysms and only mild liver involvement.

#### Literature Review (Supplementary Table S1)

Two observational studies have described MZ twins with ADPKD, both derived from the same cohort. Levy *et al.*^2^ identified 20 clinically diagnosed pairs, and Persu *et al.*^3^ reported 9 genetically confirmed pairs who had progressed to kidney failure.

Within MZ twin pairs, the difference in age at kidney failure was modest (mean: ∼ 2 years), yet significantly smaller than among nontwin siblings (mean: ∼ 7 years). The authors concluded that disease variability among MZ twins and nontwin siblings supports the role of genetic modifiers in ADPKD progression, whereas the residual intrapair differences in MZ twins are most likely due to somatic or environmental factors.

## Discussion

These data illustrate substantial phenotypic variability among MZ twins with identical *PKD1*/*PKD2* pathogenic variants, likely reflecting a combination of unidentified genetic and non-genetic influences. MZ twins, owing to early postzygotic variation events, are genetically not completely identical, differing on average by 5.2 early somatic variants.^4^ Comprehensive sequencing of ciliopathy genes in the 3 pairs identified no additional pathogenic variants or evidence of oligogenic inheritance. Both twins of pair 2 carried a heterozygous *WDR35* canonical splice variant, and both twins of pair 3 carried a heterozygous *NOS2* splice variant. These genes were previously linked to more severe disease^5^^,^^6^; however, because the detected variants were shared within pairs, they are less likely to be contributing to their phenotypic variability. Remaining possibilities include low-level somatic mosaicism or tissue-specific mosaicism (e.g., limited to kidney).^7^ Furthermore, genetic variants in regions that were not covered by the analysis (deep intronic or regulatory regions) or rare, undetected variants in genes outside known ciliopathy pathways, could still contribute to the observed differences between twins.

Epigenetic mechanisms may account for phenotypic divergence in ADPKD.^8^ Preclinical studies show that histone deacetylases, such as HDAC5, HDAC6, and SIRT1 are regulators of *PKD* genes and signaling pathways involved in cystogenesis. Other *in vitro* studies indicate that alteration of methylation patterns of the ADPKD genome and particularly in *PKD1* promoter or *PKD1* gene body may have significant impact on cystogenesis process and disease progression. Emerging evidence from miRNA studies suggests their dysregulation in ADPKD plasma and tissue samples, further supporting the role of epigenetics in modulating disease phenotype.

Environmental and comorbid influences known to accelerate ADPKD progression include arterial hypertension, urological events, obesity, high salt intake, smoking, sleep-disordered breathing, and renal injuries such as tubular crystal deposition or ischemic/nephrotoxic injury. Those with a more rapid decline in kidney function consistently exhibited ≥1 of these environmental or comorbid modifying factors. This suggests that nongenetic factors play a key role in disease progression, possibly on a similar scale as the treatment effect of tolvaptan.

Interestingly, to date, no age difference > 6 years at the occurrence of kidney failure has been reported between MZ twins, whereas differences in the age at kidney failure of > 23 years are described within families of nontwin siblings. It has been demonstrated that > 15 years difference in age at kidney failure was present in 30% of families with > 5 affected members.^9^ Thus, whereas genotype strongly determines baseline risk, environmental and stochastic factors fine-tune disease expression.

Our study has limitations. Genetic analysis cannot fully exclude mosaicism or other unrecognized variants in known or novel ciliopathy genes. Kidney tissue for epigenetic studies, including *PKD1* methylation analysis, was unavailable, as were data on 24-hour urinary sodium excretion, an established modifier of disease progression.

In summary, this study underscores the phenotypic variability among MZ twins with ADPKD. The discordance underscores that a single genetic variant is not the whole story and could be attributed to a combination of environmental factors, comorbidities, additional somatic variation in *PKD* genes, mosaicism, and epigenetic alterations, including miRNA expression. In the era of personalized medicine, accurate prognosis and management of ADPKD, even within phenotypically variable families, will probably require comprehensive genetic and nongenetic profiling of affected members.

## Disclosure

The ICMJE disclosure form for PCH has been submitted and is available from the corresponding author upon request. All other authors declared no competing interests.

## Supplementary Material

Supplementary File (PDF) **Supplementary Methods. Figure S1.** Factors likely to contribute to ADPKD clinical phenotype variability. **Table S1.** Main findings of 2 observational studies on the clinical disparities between monozygotic twins with ADPKD.
