# Supplementary material for: Clinical Variability of ADPKD in Monozygotic Twins
Source: Kidney Int Rep. 2025 Nov 11;11(2):103675. doi: 10.1016/j.ekir.2025.11.002 (PMC12769385; doi:10.1016/j.ekir.2025.11.002)
Supplement: Supplementary File (PDF) — Supplementary Methods. Figure S1. Factors likely to contribute to ADPKD clinical phenotype variability. Table S1. Main findings of 2 observational studies on the clinical disparities between monozygotic twins with ADPKD. [file mmc1.pdf]

Supplementary Methods

Study Population

All procedures were approved by the local ethics committee of UZ Brussel. Pairs of MZ twins were identified from the outpatient ADPKD clinic of UZ Brussel. After obtaining informed consent (B.U.N.143201524795), clinical and demographic data were recorded from their first visit until they developed kidney failure. Averaged values for BMI, blood pressure, and kidney function were calculated for each year of age based on recorded data.

The diagnosis of ADPKD was genetically confirmed in all participants.

The diagnosis of monozygosity was based on physical resemblance and the percentage of population SNPs in common. This percentage was confirmed as 99% or more.

Genetic Analysis

Molecular testing of *PKD1* and *PKD2* was performed by targeted next generation sequencing in all patients, utilizing a custom gene panel (SureSelect, Agilent) containing coding regions ± 50 bp flanking intronic regions with either 136 or 356 known or candidate PKD and ciliopathy genes as described by *Hopp et al., Kidney Int. 2020;97:370–382*. The libraries were prepared using the NEBNext Ultra DNA preparation kit, where samples were indexed with dual-index primers and libraries of 24 samples were pooled before capture. Sequencing was performed on the Illumina NovaSeq platform, generating 150bp paired end reads. Panel analysis was performed using SVS Golden Helix Software with the following filtering thresholds: variant locus read-depth >10 and genotype quality >20; GnomAD Exome, GnomAD Genome, and ExAC Exome minor allele frequency all ≤1.0. The remaining variants were individually evaluated for pathogenicity based on: predicted loss of function (nonsense, frameshift, canonical splice variants); inclusion in the online ADPKD database (pkdb.mayo.edu); ACMG classified as pathogenic or likely pathogenic (Franklin); previously reported as pathogenic or likely pathogenic in ClinVar and HGMD; multisequence alignment; and predicted to alter splicing (Berkeley Drosophila Genome Project

and SpliceAI). Large copy number variants (CNVs) were assessed by calculating the log2 ratio of actual read-depth over expected read-depth for a given locus. Variants with log2 ratios greater than 0.5 or less than -0.75 were considered CNV candidates. BAM files were reviewed to confirm coverage of targeted genes, zygosity and read depth of identified variants, and to identify CNV breakpoints when possible. No significant CNV were detected for any patients.

Search Strategy

We searched the MEDLINE database on 20th March 2025, without time limit, using the terms *(“Autosomal Dominant Polycystic Kidney Disease” OR “Polycystic Kidney Disease” OR “Polycystic Kidney, Autosomal Dominant”[Mesh]) AND (“Identical Twins” OR “Monozygotic Twins” OR “Twins, Monozygotic”[Mesh])*. Reference lists of original articles were searched for further eligible articles.

Statistical analysis

For twin pairs 1 and 2, linear regression of estimated glomerular filtration rate chronic kidney disease epidemiology collaboration (eGFR CKD-EPI) as a function of age was performed for each individual, with missing values imputed by linear interpolation.

The mean, maximum and minimum decline in eGFR CKD-EPI was calculated for each individual.

Supplementary Genetic Results

In Twin Pair 1, the *PKD1* variant (c.2249G>A, p.Trp750Ter) was detected that was not found in GnomAD Exomes or Genomes, and was classified Likely Pathogenic by ACMG (PVS1, PM2). None of the pseudogene sequences contain threonine at this position. In Twin Pair 2, the detected *PKD1* variant (c.11343C>G, p.Tyr3781Ter) was not found in GnomAD Exomes or Genomes, and was classified Pathogenic by ACMG (PVS1, PM2, PS4). This variant is located outside the pseudogene-duplicated region in *PKD1*. In Twin Pair 3, the variant *PKD2* (c.2635del, p.Glu879ArgfsTer30) was not found in GnomAD Exomes or Genomes, and was classified Likely Pathogenic by ACMG (PVS1, PM2). BAM review showed a read depth of greater than 200 at each locus, with the proportion of alternate allele reads between 40-59% for each variant.

Supplementary Figures and Tables

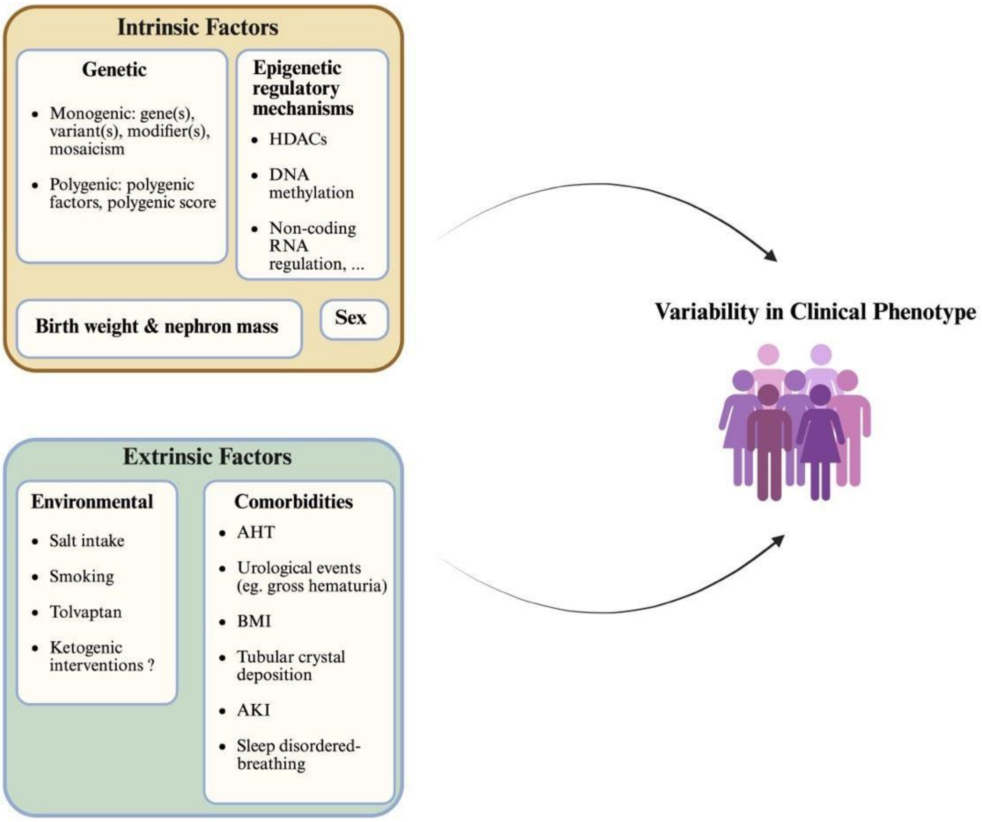

Figure S1: Factors likely to contribute to ADPKD clinical phenotype variability. HDACs, histone deacetylases; DNA, deoxyribonucleic acid; miRNA, micro ribonucleic acid; AHT, arterial hypertension; BMI, body-mass index; AKI, acute kidney injury

		Levy et al, 1995	Persu et al, 2004
**Subjects**			
	Monozygotic twin pairs	20	9
	Female/Male	11/9	3/6
	Age range at diagnosis (years)	7 – 52	N/A
	Age difference at diagnosis, in years (N)	0 (9)<br>< 2 (3)<br>> 2 (8)	N/A
**Hypertension**			
	Monozygotic twin pairs (%)	14/20 (70%)	N/A
	Age difference at diagnosis, in years (N)	0 (3)<br>< 3 (3)<br>3 – 19 (8)	N/A
	Mean age difference at diagnosis (years)	5.6	N/A
	Age difference at treatment, in years (N)	0 (1)<br>< 3 (3)<br>3 – 16 (10)	N/A
	Mean age difference at treatment (years)	4.7	N/A
**Kidney failure**			
	Monozygotic twin pairs (%)	7/20 (35%)	9/9 (100%)
	Age range at kidney failure (years)	36 - 54	36 - 59
	Mean age at kidney failure (years)	N/A	46.8 ± 7.4
	Age difference at kidney failure, in years (N)	< 1.5 (3)<br>1.5 – 6 y (4)	0.1 - 6 (9)
	Mean age difference at kidney failure (years)	2.1	2.1 +/- 1.9

Table S1: Main findings of two observational studies on the clinical disparities between monozygotic twins with ADPKD. ADPKD, Autosomal Dominant Polycystic Kidney Disease; N/A, not available; N, number of twin pairs
